# Mreg Activity in Tumor Response to Photodynamic Therapy and Photodynamic Therapy-Generated Cancer Vaccines

**DOI:** 10.3390/cancers8100094

**Published:** 2016-10-15

**Authors:** Mladen Korbelik, Judith Banáth, Wei Zhang

**Affiliations:** British Columbia Cancer Agency, Vancouver, BC V5Z 4E6, Canada; jbanath@bccrc.ca (J.B.); wzhang@bccrc.ca (W.Z.)

**Keywords:** photodynamic therapy, cancer vaccines, Mregs, anti-GR1 antibody

## Abstract

Myeloid regulatory cells (Mregs) are, together with regulatory T cells (Tregs), a dominant effector population responsible for restriction of the duration and strength of antitumor immune response. Photodynamic therapy (PDT) and cancer vaccines generated by PDT are modalities whose effectiveness in tumor destruction is closely dependent on the associated antitumor immune response. The present study investigated whether the immunodepletion of granulocytic Mregs in host mice by anti-GR1 antibody would improve the response of tumors to PDT or PDT vaccines in these animals. Anti-GR1 administration immediately after Temoporfin-PDT of mouse SCCVII tumors abrogated curative effect of PDT. The opposite effect, increasing PDT-mediated tumor cure-rates was attained by delaying anti-GR1 treatment to 1 h post PDT. With PDT vaccines, multiple anti-GR1 administrations (days 0, 4, and 8 post vaccination) improved the therapy response with SCCVII tumors. The results with PDT suggest that neutrophils (boosting antitumor effect of this therapy) that are engaged immediately after photodynamic light treatment are within one hour replaced with a different myeloid population, presumably Mregs that hampers the therapy-mediated antitumor effect. Anti-GR1 antibody, when used with optimal timing, can improve the efficacy of both PDT of tumors in situ and PDT-generated cancer vaccines.

## 1. Introduction

The field of cancer immunotherapy has made highly important progress during the past decade, particularly by developing a number of potent antitumor immunity activation protocols [1]. However, lasting therapeutic responses were still remaining largely unattainable and it was only recently established that the attainment of complete tumor elimination requires maintaining the mobilized tumor-specific T cells and immune effectors in the activated state by preventing that they are rendered tolerant or exhausted [2]. Hence it has become clear that for achieving a decisive success with cancer immunotherapy it is necessary to relieve the inhibitory effects of immunoregulatory mechanisms (developed to assure self-tolerance) that are manifested as either immunoregulatory cell activity or through inhibitory intercellular signals mediated by multiple negative checkpoint regulators [3,4].

Among various immunoregulatory cell types, dominant roles are played by lymphoid CD^4+^CD^25+^ T cell population (Tregs) and myeloid regulatory cells (Mregs) also known as myeloid-derived suppressor cells (MDSCs) [5,6]. It has become evident that Mregs consist of a heterogeneous mixture of myeloid cells at different stages of differentiation that suppress CTL and Th1 responses in malignant lesions [7]. We have recently shown that Mregs, together with Tregs, have a critical negative impact on therapy outcome with photodynamic therapy (PDT)-generated cancer vaccines [8]. While PDT is clinically established treatment for malignant and other diseases based on the use of visible light for activating photosensitizing drugs to produce cytotoxic reactive oxygen species [9], its exploitation for creating therapeutic cancer vaccines remains in pre-clinical stage of investigation [10]. In contrast to standard PDT where tumors are illuminated in situ, with PDT vaccine tumor tissue or derived cells are exposed to light ex vivo to generate therapeutic autologous whole-cell vaccines effective in generating immune response tailored against individual patient’s malignancy [10,11].

In the presented study, we investigated the use of anti-mouse GR1antibody produced by hybridomas clone RB6-8C5 in elucidating the role of granulocytic myeloid populations in therapeutic outcome of tumor treatment by PDT and PDT vaccines. This antibody binds to mouse myeloid differentiation antigen and is known to effectively deplete granulocytes from mice [12]. The results indicate that GR1 treatment can deplete not only mature neutrophils but also tumor-sensitized granulocytic Mregs and can drastically alter therapy outcome after PDT or PDT vaccine application.

## 2. Results and Discussion

**Figure 1:**
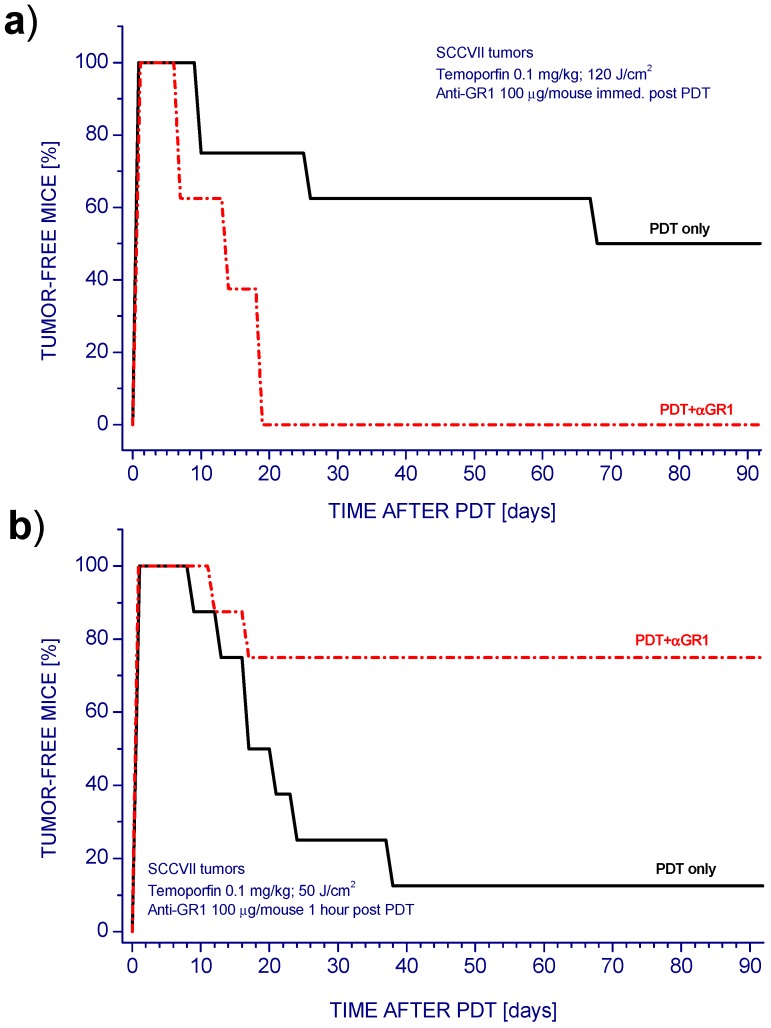
The effect of anti-GR1 antibody on photodynamic therapy (PDT) response of SCCVII tumors. Mice bearing SCCVII tumors were administered Temoporfin (0.1 mg/kg i.p.) and 24 h later the tumors were illuminated with 650 ± 10 nm light. Anti-GR1 was given i.v. at the dose of 0.1 mg/mouse. (**a**) PDT light dose 120 J/cm^2^ and anti-GR1 injected immediately after light treatment; (**b**) PDT light dose 50 J/cm^2^ and anti-GR1 injected 1 h after light treatment. Mice (8 per group) were monitored up to 90 days for tumor growth. There were statistically different responses (*p* < 0.05) for PDT alone compared to PDT plus anti-GR1 groups in both graphs.

We have used the anti-GR1monoclonal antibody RB6-8C5 in a number of our PDT studies employing various mouse tumor models [13,14,15]. This antibody is particularly effective in rapidly (largely within 15 min) depleting neutrophils from murine peripheral blood and various organs [15]. Neutrophils were suggested to directly affect post-PDT T-cell proliferation and antitumor responses mediated by cytotoxic T-lymphocytes [16]. In our PDT studies with various mouse tumor models, anti-GR1 was routinely applied immediately after tumor PDT light treatment by iv. injection of the antibody into host mice. As shown with SCCVII tumors treated by Temoporfin-PDT, this renders a largely curative PDT protocol ineffective, since there was recurrence of all tumors after their initial ablation (Figure 1a). This finding is in the accordance with our results with different photosensitizers and tumor models used for PDT and with reports from other researchers [13,17]. In striking contrast, delaying anti-GR1 injection from 0 to 60 min post PDT produced an opposite effect. It converted PDT treatment of SCCVII tumors that was poorly curative to highly curative (Figure 1b). It should be mentioned that no modulations in tumor response to PDT were observed when anti-GR1 antibody was substituted with its isotype control [18].

The remarkable turnabout produced by delaying anti-GR1 injection suggests that within a relatively short time interval after PDT a different GR1-sensitive cell population takes over from inflammatory neutrophils the decisive control of tumor response. Neutrophils are well recognized as responsible for the early effect, because they rapidly accumulate in PDT-treated tumors in large numbers and become engaged as activated inflammatory effectors releasing reactive oxygen metabolites and a variety of toxic agents capable of inflicting a pervasive damage of tumor tissue [19,20]. Therefore, it is not surprising that the GR1-mediated immunodepletion of these cells in host mice immediately after reduces the efficacy of tumor PDT. On the other hand, myeloid cells targeted by anti-GR1 injection 1 h later should be engaged in hindering tumor response to PDT since their elimination improves the therapeutic efficacy of PDT. Hence, activated inflammatory neutrophils extravasated into tumor tissue within minutes after PDT appear to be replaced by a different population of myeloid cells. In fact, granulocytic Mregs were recently defined as one of multiple phenotypes of neutrophils, i.e., a subpopulation of neutrophils with specialized function [21]. Thus, it seems possible that within the first hour after PDT such functional subset of neutrophils is generated by phenotype switching or priming directed by damage-associated molecular patterns (DAMPs) or other signals released from PDT-treated tumor.

**Figure 2:**
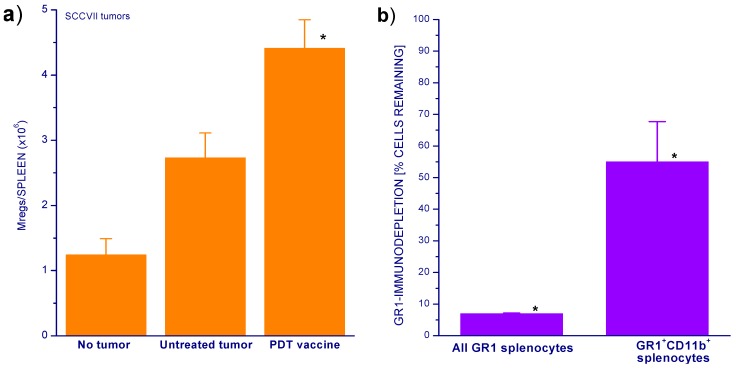
Spleen Mreg levels in untreated and PDT vaccine treated mice and the impact of anti-GR1 mediated immunodepletion. (**a**) Spleens were collected 72 h after PDT vaccine treatment (SCCVII cells treated with ce6-PDT, post-incubated 16 h, and injected at 2 × 10^7^/mice as described in Experimental Section). Splenocytes from these spleens and from control untreated mice were stained for GR1 and CD11b markers for determining granulocytic Mreg levels by flow cytometry; (**b**) mice were injected i.v. with anti-GR1 antibody (0.1 mg per mouse) and sacrificed 24 h later for determining the numbers of remaining GR1-positive splenocytes and granulocytic Mregs (GR1^+^CD11b^+^) by antibody staining followed by flow cytometry. *n* = 4, bars are SD. * Statistically significant difference (*p* < 0.05) from control groups or pre-treatment levels.

Our recent findings reveal that the incidence of myeloid cells dominated by granulocytic Mregs and their activation state are elevated in mice bearing SCCVII tumors treated by PDT vaccines [8]. The numbers of these cells (identified by a highly positive staining for markers GR1 and CD11b) found in the spleens were increased already in mice bearing untreated tumors but were even more dramatically higher after PDT vaccine treatment (Figure 2a). The examination of the impact of GR1-mediated immunodepletion treatment of SCCVII tumor-bearing mice shows that this treatment was effective in eliminating not only the majority of general GR1^+^ splenocytes population (dominated by neutrophils) but also about the half of Mregs found in the spleen (Figure 2b). Thus, the effect of GR1 immunodepletion on response of PDT-treated tumors documented in Figure 1b could be attributed to restricting the inhibitory activity of Mregs.

**Figure 3:**
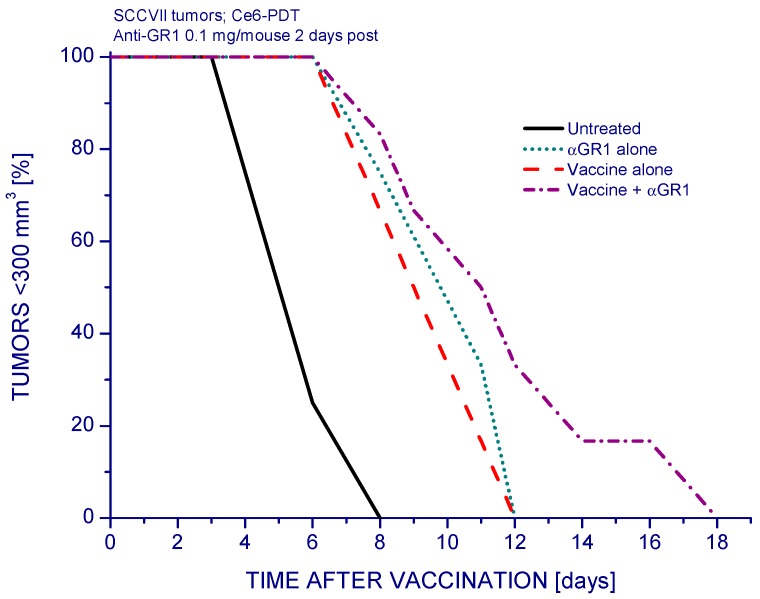
The effect of anti-GR1 treatment on the response of SCCVII tumors to PDT vaccine. Two days after peritumoral administration of PDT vaccine (prepared as described for Figure 2), the mice were injected with anti-GR1 (0.1 mg/mouse i.v.) or control saline. Control groups include mice treated with anti-GR1 alone. The response to therapy was evaluated by tumor size measurement and is presented as percentages of mice with tumors smaller than 300 mm^3^ in function of time after vaccination. Each treatment group consisted of 6 mice. Statistically significant (*p* < 0.05) were the differences between the control untreated group response and all the three treated group responses.

Based on the above findings it can be suggested that the anti-GR1 antibody can (in appropriately designed protocols) serve with mouse tumor models, and its human counterpart with clinical cancer therapy protocols, as an adjuvant to therapies whose effectiveness is reduced by the activity of Mregs. Indeed, several groups have tested the effects of RB6-8C5 antibody administration with mouse tumor models aiming at the depletion of GR1-positive Mregs demonstrating that it restores the antitumor T cell-mediated responses and preventing tumor relapse [22,23]. In addition to examining the PDT of tumors in situ, we examined the effectiveness of anti-GR1 treatment as adjuvant to PDT vaccines using the protocol standardized in our previous studies [24]. The PDT vaccine treatment alone resulted in significant tumor growth retardation (Figure 3). Single anti-GR1 antibody injection alone also produced a similar effect, but when administered 2 days after the vaccine treatment it produced a further retardation of growth of vaccinated tumors although the therapeutic impact compared to the result with PDT vaccine alone was not statistically significant.

**Figure 4:**
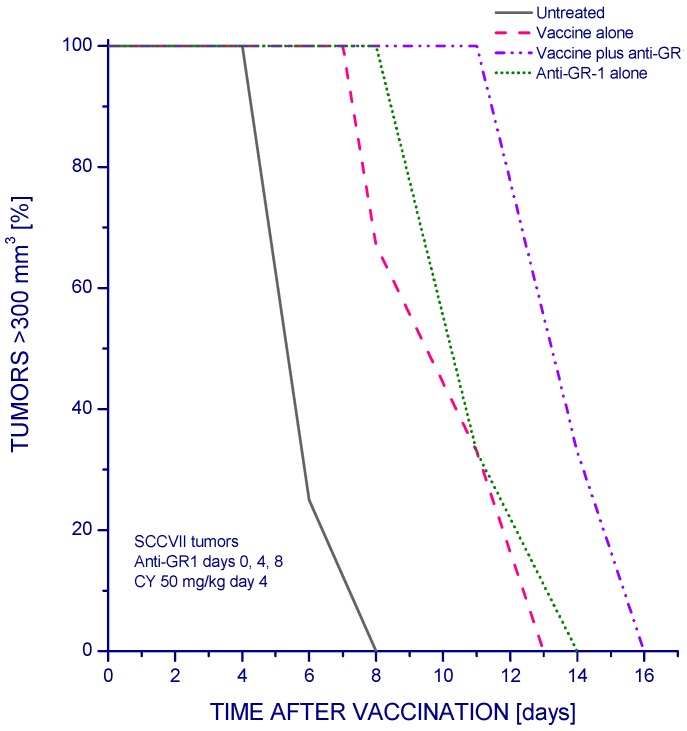
The effect of multiple anti-GR1 treatment on the response of SCCVII tumors to PDT vaccine. The mice received PDT vaccine treatment as described for Figure 3 but were also administered cyclophosphamide (50 mg/kg i.p.) at day 4 post vaccination. Anti-GR1 (0.1 mg/mouse i.v.) was injected on days 0, 4, 8 relative to the time of vaccination. The response to therapy was evaluated by tumor size measurement and is presented as percentages of mice with tumors smaller than 300 mm^3^ in function of time after vaccination. Each treatment group consisted of 6 mice. Statistically significant (*p* < 0.05) were the differences between the control untreated group response and all the three treated group responses, and between vaccine alone and vaccine plus anti-GR1 group.

Since Mregs levels in PDT vaccine-treated mice that were reduced after anti-GR1 injection can be replenished within several days [15,23], we tested the effect of anti-GR1 injected on 0, 4, and 8 days relative to PDT vaccine application. In addition, low-dose cyclophosphamide was included in PDT vaccine protocol for blocking the activity of Tregs as practiced in our previous work [8]. The results show that the therapeutic efficacy of PDT vaccine treatment (that significantly delayed SCCVII tumor growth) was further improved in the group receiving injections of anti-GR1 (Figure 4). Comparable injections of isotype control antibody had no therapeutic impact [25]. Comparison between Figure 3 and Figure 4 suggests that the inclusion of cyclophosphamide treatment had in this case only a minor impact. Hence, the benefit seen with the vaccine plus anti-GR1 group in Figure 4 could be attributed to the prolongation of anti-GR1 treatment.

Dampening immunosuppressive actions of Tregs and Mregs is now considered a key approach for securing a successful outcome of tumor immunotherapy [3]. A number of strategies for restraining Mreg activity have been tested in pre-clinical and clinical studies [26]. We have shown that at least Mreg-depleting agents, all-*trans* retinoic acid (ATRA) [8] and anti-GR1 antibody (this work) have the potential to improve the response to tumor therapy mediated by PDT vaccines. Each of these two has possible setbacks. For ATRA, it was found that it can promote Treg activity [27]. With GR1 depletion, there is a concern of side-effects caused by temporary elimination of granulocytes rendering the host vulnerable to opportunistic infections [28] but our results suggest that in some cases prolonged injecting of this antibody may not be necessary. Since there are suggestions that the RB6-8C5 antibody can also bind to monocytic (Ly6C) populations, although this has been disputed [29], a possibility of anti-GR1 treatment affecting not solely granulocytic but also monocytic Mreg subpopulations cannot be excluded. However, no GR1-mediated immunodepletion of monocytic populations was detected in our studies with mouse models. It should be also mentioned that in this paper the granulocytic Mregs have not been characterized with respect to their cytokine profile and other functional attributes. Recently, we have reported on another interesting agent, acid ceramidase inhibitor LCL521, which inhibits the engagement of both Tregs and Mregs and acts as an effective adjuvant to PDT vaccines [30].

## 3. Materials and Methods

### 3.1. Tumor Model

The experimental procedures involving mice were approved by the Animal Care Committee of the University of British Columbia. The tumor model used, SCCVII squamous cell carcinoma is a well recognized head and neck cancer model of spontaneous origin with limited immunogenicity [31]. It was grown in 7–9 weeks old syngeneic C3H/HeN mice by inoculating subcutaneously 1 × 10^6^ SCCVII cells into depilated lower dorsal region.

### 3.2. PDT and PDT Vaccine Treatments

Treatment by PDT of tumors in situ was performed by injecting Temoporfin (0.1 mg/kg i.p.) into host mice 24 h before tumor illumination (650 ± 10 nm with 80–90 mW/cm^2^ fluence rate). Use of different PDT light doses in Figure 1a,b was necessary for adequately capturing tumor cure changes produced by varied anti-GR1 treatment time. The photosensitizer Temoporfin (Foscan) was provided by Biolitec Research GmbH (Jena, Germany). Other details of light delivery were described earlier [32]. At chosen intervals after PDT, some mice received 0.1 mg i.v. injection of GR1 antibody (rat IgG2b obtained from RB6-8C5 hybridoma) or rat IgG2b from clone 2F8 (recognizing mouse scavenger receptor) used as an isotype control antibody. After therapy, the mice were monitored for signs of tumor growth and no sign of tumor at the end of observation period (90 days) qualified as a cure.

For treatment of tumors by PDT vaccine, SCCVII cells were exposed to PDT in vitro by incubating them first 30 min with 1 µg/mL concentration of photosensitizer chlorin e6 (ce6, produced by Frontier Scientific, Logan, UT, USA). Next, the cells were treated by 665 ± 10 nm light (1 J/cm^2^; 30 mW/cm^2^) and then left in culture for 16 h in Ex-cell NS0 serum-free medium (Sigma Chemical Co., St. Louis, MO, USA). The cells were then collected, exposed to X-rays (60 Gy), and delivered (2 × 10^7^ per mouse) by peritumoral injection. Further details of experimental protocol were specified elsewhere [24,32]. Cyclophosphamide (Sigma) administration, 50 mg/kg dose i.p. at 4 days after PDT vaccine, was included in some experiments. Post-vaccination treatment with anti-GR1 or its isotype control consisted of single or multiple injections of the same dose as used for PDT in situ.

### 3.3. Flow Cytometry

For granulocytic Mreg level assessment, splenocytes released from spleens collected from mice at 3 days after PDT vaccine treatment or control untreated mice were stained with phycoerythrin-cyanine5-conjugated rat anti-mouse GR1 (eBioscience, San Diego, CA, USA) and phycoerythrin-conjugated anti-mouse CD11b produced in mouse (Santa Cruz Biotechnology Inc., Dallas, TX, USA). For GR1-mediated immunodepletion, the antibody was administered at 0.1 mg/mouse i.v. 24 h before the spleen collection from sacrificed mice. The splenocyte suspensions were then analyzed by flow cytometry using a Coulter Epics Elite ESP (Coulter Electronics, Hialeah, FL, USA), with 2 × 10^4^ cells included in each test. The (total) number of Mreg cells/spleen was derived from the yields of GR1^+^CD11b^+^ cells from known weights of tissue. The percentage of GR1-immunodepleted cells was calculated from total cell numbers.

### 3.4. Statistical Analysis

Log-rank test was used for statistical evaluation with survival-type tumor responses, while Mann-Whitney test served for the analysis of other data. The threshold for statistical significance was set at 5%.

## 4. Conclusions

Immunodepletion of myeloid populations targeted by anti-GR1 antibody, when conducted at the right timing, augmented cures of host mice that can be attributed to blocking the activity of Mreg associated with responses to tumor treatment by PDT or PDT-generated cancer vaccines.
